# Genetic Diversity and Differentiation Pattern of *Mastacembelus armatus* in the Dongjiang and Ganjiang River Sources

**DOI:** 10.3390/biology15110869

**Published:** 2026-05-31

**Authors:** Bin Wu, Yuan Fang, Qingxiang Zeng, Han Li, Yanping Zhang, Haihua Wang

**Affiliations:** 1 Jiangxi Fisheries Research Institute, Nanchang 330039, China; wubinjx@163.com (B.W.); lh_jxsks@163.com (H.L.); 2 Ganzhou Animal Husbandry and Fisheries Research Institute, Ganzhou 341000, China; shiqusu@163.com (Y.F.); gnasskszqx@163.com (Q.Z.)

**Keywords:** zig-zag eel, genome resequencing, population structure, genetic divergence, adaptive evolution

## Abstract

To explore the genetic diversity and evolutionary differentiation of *Mastacembelus armatus* from the headwaters of the Dongjiang and Ganjiang Rivers, we performed whole-genome resequencing on three populations, including Xunyushui (XW) and Jiuqu River (DN) from the Dongjiang River source as well as Taojiang River (XF) from the Ganjiang River source. We analyzed population structure, genetic differentiation, nucleotide diversity (π), pairwise FST, linkage disequilibrium, kinship, and neutrality tests (Tajima’s D, Fu and Li’s D). A total of 209.05 Gbp of clean data were obtained, with high quality and reliable alignment. Average nucleotide diversity (π) was higher in XW (0.00490 ± 0.00248) and DN (0.00478 ± 0.00312) and lower in XF (0.00463 ± 0.00158). Pairwise FST values revealed moderate differentiation between XW and DN (FST = 0.12) and strong divergence between XF and the other two populations (FST = 0.19 and 0.17). Neutrality tests showed no significant deviation from neutrality. XW and DN exhibited positive values, indicating stable demography, while XF showed negative values, suggesting a tendency of population expansion. Phylogenetic, admixture, and PCA analyses supported that all three populations belonged to one evolutionary clade with two ancestral components. XF showed the slowest linkage disequilibrium decay and distant kinship, indicating a small effective population size. Significant genetic divergence was primarily driven by geographic isolation and limited gene flow. This study reveals the genetic diversity and differentiation pattern of *M. armatus* and provides a genomic basis for its conservation and management.

## Simple Summary

Wild zig-zag eel (*Mastacembelus armatus*) populations are declining due to environmental damage and overfishing, making their conservation intervention urgent. This study used whole-genome resequencing to examine the genetic diversity and adaptation of three zig-zag eel groups from the Dongjiang River source and Ganjiang River source in southern China. We found that all three groups belong to the same evolutionary lineage but show clear genetic differences. The Xunwushui and Jiuqu River groups have high genetic diversity and close genetic relationships, while the Taojiang River group has lower genetic diversity and distinct genetic features. These differences are mainly caused by geographic isolation and limited gene flow between river systems. Our results provide genome-level guidance for protecting the genetic resources of zig-zag eels, developing effective conservation plans, and supporting the sustainable management of this important freshwater fish.

## 1. Introduction

*Mastacembelus armatus* (Lacepède, 1800), belonging to the genus *Mastacembelus*, family Mastacembelidae, order Synbranchiformes, is an economically important freshwater fish in southern China with high edible, nutritional and ecological value [1]. In recent years, its wild populations have declined sharply due to aquatic ecological degradation, overfishing and other anthropogenic disturbances. Fishery resource surveys were conducted in collaboration with Ganzhou Animal Husbandry and Fisheries Research Institute and Jiangxi Fisheries Research Institute to investigate the relative population abundance and dominance of *M. armatus* in the Jiuqu River and Taojiang River. The results showed that in the Jiuqu River, 62 individuals were collected, accounting for 19.44% of the total catch and 17.18% of the total biomass, with an IRI value of 0.3662, ranking second among all fish species and being a dominant population. In the Taojiang River, 18 individuals were collected, accounting for 6.45% of the total catch and 14.23% of the total biomass, with an IRI value of 0.2083, ranking third and also being a dominant population. Comparative analysis indicated that the populations of *M. armatus* in both rivers have shown a declining trend in abundance and miniaturization in body size in recent years. As *M. armatus* has been listed as a provincial key protected wild animal in Guangdong, Fujian and other provinces, the conservation and sustainable utilization of its germplasm resources have become a key research focus in aquaculture and aquatic biodiversity conservation [2,3].

To promote the resource conservation and industrial application of *M. armatus*, researchers have conducted systematic studies across multiple disciplines, including cross breeding, growth regulation, nutritional feed, disease immunity, sex determination and differentiation, and population genetics. Notably, studies on heterosis in the context of distant hybridization have provided valuable insights into the germplasm improvement of this species; however, this research direction primarily centers on cross breeding and heterosis mechanisms, which differs distinctly from the population genetics-oriented focus of the present study. Existing cross breeding research has preliminarily explored the mechanism of heterosis in distant hybridization as a core strategy for germplasm enhancement: Chen et al. [1] verified that phenotypic differentiation of hybrid progeny from *M. armatus* and *M. lateralis* is closely associated with differentially expressed genes in key pathways such as the insulin-like growth factor (IGF) signaling pathway and skeletal muscle development. Yang et al. [4] combined transcriptomic and metabolomic analyses to clarify the correlation between heterosis in hybrid offspring and pathways regulating growth and material metabolism. Sun et al. [5] revealed that disordered expression of NAD(P)H-related genes accounts for the low hatching rate and high malformation rate in hybrids of *M. armatus* and *Sinobdella sinensis*, offering a reference for hybrid parental selection. In contrast, research on growth regulation has laid a foundation for molecular-assisted breeding: Han et al. [6] identified multiple growth-associated SNP loci and candidate genes via a genome-wide association study (GWAS), while Luo et al. [7] developed 42 microsatellite markers to support molecular marker-assisted selection. Xue et al. [8] and Zhong et al. [9] further dissected the regulatory roles of the growth hormone/insulin-like growth factor 1 (GH/IGF-1) axis and growth hormone-releasing hormone (GHRH). Additionally, Xue et al. [10] demonstrated that hydrolysable tannin can improve the growth performance of juvenile *M. armatus*, optimizing feed formulation strategies for this species.

Population genetic diversity and population structure constitute the fundamental basis for species resource conservation. Previous population genetic studies on *M. armatus* have been carried out using low-throughput molecular tools such as mitochondrial genomes and microsatellite markers. Wang et al. [11] uncovered the population structure and historical dynamics of *M. armatus* in southern China via mitochondrial genome analysis, confirming the driving effect of geographical isolation on population differentiation. Tingting et al. [2] further verified the impact of river system patterns on the genetic structure of *M. armatus* populations using microsatellite markers. Wu et al. [3] provided data support for regional conservation efforts through resource assessment of *M. armatus* populations in the Taojiang River, Jiangxi Province. In the field of reproductive biology, Alam et al. [12] and Rashid et al. [13] supplemented basic growth and reproductive data for different geographical populations, while Moosa et al. [14] provided warnings for wild population health management through pathogen detection.

Nevertheless, current research on *M. armatus* has notable limitations. First, population genetic analyses based on low-throughput molecular markers fail to comprehensively characterize genome-wide genetic variation. Second, systematic genomic studies on key geographical populations, such as those from the Dongjiang River source and Ganjiang River source, are lacking, impeding the implementation of regional precise conservation. Third, the molecular mechanisms underlying environmental adaptive evolution remain poorly understood, restricting the efficient utilization of genetic resources. To address these gaps, this study selected three representative *M. armatus* populations from the Dongjiang River source (Xunwushui, XW; Jiuqu River, DN) and Ganjiang River source (Taojiang River, XF) for whole-genome resequencing. We systematically analyzed the population genetic structure, differentiation characteristics and selection signals of these populations, clarified the discrepancies in genetic diversity and evolutionary patterns between *M. armatus* populations from the two river sources, and identified candidate selected genes associated with environmental adaptation. The findings of this study not only provide genomic resources for evaluating genetic diversity and developing functional trait molecular markers of *M. armatus*, but also lay a scientific foundation for regional population conservation, germplasm resource exploration and sustainable utilization of this species.

## 2. Materials and Methods

### 2.1. DNA Extraction

Basic information of sampled *M. armatus* individuals collected from 26 to 28 July 2021 is presented in Table 1, with 10 individuals sampled per population. The muscle samples were immediately preserved in liquid nitrogen on site, and genomic DNA was extracted from the muscle tissues using the Marine Animal Tissue Genomic DNA Extraction Kit (DP324, Tiangen Biotech Co., Ltd. Beijing, China). After extraction, the quality of the isolated DNA was detected by 1.2% agarose gel electrophoresis (Bio-Rad Laboratories, Inc., Hercules, CA, USA), and the concentration and purity of DNA were determined using a Nanodrop D2000 ultramicro spectrophotometer (Thermo Fisher Scientific, Wilmington, DE, USA). This study was approved by the Institutional Animal Care and Use Committee (IACUC) of Jiangxi Fisheries Research Institute. All experimental procedures were strictly performed in accordance with the ethical norms and the formulated rules and regulations of Jiangxi Fisheries Research Institute.

### 2.2. Sample Sequencing and Sequence Alignment

Samples were sent to Beijing Biomarker Technologies Co., Ltd. for sequencing. Genomic DNA was randomly sheared using a Covaris M220 focused-ultrasonicator. The sonication parameters were set as follows: peak power of 140 W, duty factor of 10%, 200 cycles per burst, and a treatment duration of 60–75 s. After ultrasonic fragmentation, the sheared DNA was purified, followed by end repair, 3′-end adenylation, and sequencing adapter ligation. Target DNA fragments were further selected via agarose gel electrophoresis, and PCR amplification was performed to construct the final sequencing library. All libraries were strictly quality-checked before high-throughput sequencing on the Illumina platform (Illumina, Inc., San Diego, CA, USA).

The raw sequenced reads obtained from whole-genome resequencing inevitably contained adapter sequences and low-quality reads. To ensure the validity and credibility of subsequent bioinformatics analysis, raw reads were preprocessed and filtered to generate high-quality clean reads for further downstream analysis.

The detailed filtering criteria were as follows:

(1) Reads contaminated with adapter sequences were removed;
(2) Reads with an ambiguous base (N) proportion exceeding 10% were discarded;
(3) Reads with over 50% of bases possessing a Phred quality value lower than Q10 were eliminated.

The clean reads were aligned against the *M. armatus* genome [15] using BWA software (v. 0.7.17, Heng Li, Boston, MA, USA) [16]. Samtools [17,18] was employed to calculate the alignment rate and coverage.

### 2.3. Variant Detection and Structural Annotation

The detection of single nucleotide polymorphism (SNP) and small insertion and deletion (small InDel) was performed using the GATK software package (version 4.2) [19]. Based on the mapping results of clean reads aligned to the reference genome, samtools (v1.9) [18] was used to mark and filter redundant duplicate reads to ensure the accuracy of subsequent variant detection. The HaplotypeCaller algorithm embedded in GATK was adopted to detect SNP and small InDel variants. The gVCF file of each sample was generated independently in the initial step, followed by population joint genotyping analysis. Finally, strict variant filtering was conducted to obtain the final set of valid variant loci.

Strict filtering was performed on the raw variant detection results to guarantee the reliability and accuracy of the obtained variants. The main filtering criteria were set as follows:

(1) The subsidiary program vcfutils.pl (varFilter -w 5 -W 10) in bcftools was used to filter SNPs within 5 bp flanking regions of InDels, as well as InDels located within 10 bp adjacent to other InDels.
(2) The parameter settings of clusterSize 2 and clusterWindowSize 5 were applied, which meant that the number of variant loci within a 5 bp sliding window should not exceed 2.
(3) Variants with QUAL values lower than 30 were removed. The QUAL value is a Phred-scaled quality score representing the confidence of variant existence at the corresponding locus.
(4) Variants with QD values lower than 2.0 were discarded. QD refers to the ratio of variant quality score to sequencing depth, where the sequencing depth is the total coverage depth of all samples carrying variant bases at this locus.
(5) Variants with MQ values lower than 40 were filtered out. MQ represents the root-mean-square mapping quality of all reads mapped to the corresponding variant locus.
(6) Variants with FS values higher than 60.0 were eliminated. The FS value is converted from the *p*-value of Fisher’s exact test, which evaluates the significant strand bias between variant reads and reference reads during sequencing and alignment. A reliable variant without strand-specific bias should have an FS value close to zero.
(7) Other variant filtering parameters followed the default official thresholds of GATK.

ANNOVAR (version 2020Jun08) [20] and snpEff (version 5.0) [21] software were utilized to perform functional and positional annotation for the SNPs and INDELs.

### 2.4. Population Evolutionary Analysis

Only the genome resequencing data of 30 wild *M. armatus* individuals sampled in this study was used for phylogenetic analysis. No additional public reference sequences or sequences from other studies were adopted. The final alignment length for phylogenetic construction was 823,658 bp. The phylogenetic tree was constructed using MEGA X software based on the neighbor-joining (NJ) method. The Kimura 2-parameter evolutionary model was selected, and bootstrap analysis with 1000 replicates was performed to evaluate the topological reliability of the tree [22]. Principal component analysis (PCA) was performed using the EIGENSOFT software [23] based on the acquired SNP data to investigate the genetic clustering patterns among all samples. PCA can effectively reflect the genetic similarity and divergence among individual samples and further identify genetically close and distant relationships across populations, which provides reliable auxiliary evidence for subsequent phylogenetic evolutionary analysis.

The population genetic structure of the studied populations was analyzed using the admixture software [24] based on the screened SNP markers. In this analysis, the putative number of genetic subgroups (K value) was preset to range from 1 to 10 for cluster analysis. Cross-validation was further performed on all clustering results, and the optimal K value for population grouping was determined according to the minimum cross-validation error rate. The GCTA software [25] was used to estimate the pairwise genetic relatedness among individuals in the natural population. Genetic relatedness matrices mainly include the A matrix and the G matrix. The A matrix is generally calculated based on pedigree information, while the G matrix is constructed using genomic molecular markers, namely SNP markers in this study. In the present analysis, the G matrix was adopted, with the expected variance of markers corrected by the mean value of markers’ expected variance by default. LD decay analysis was performed using PLINK2 [26] to calculate the linkage disequilibrium (LD) between pairwise SNPs on the same chromosome within a physical distance of 1000 kb. The intensity of linkage disequilibrium was quantified by the coefficient of determination r^2^. A value of r^2^ closer to 1 indicates stronger linkage disequilibrium. Genome-wide detection of selective sweep regions is conducted by calculating population genetic indicators of all SNP loci within sliding windows (e.g., 100 kb) with a fixed step size (e.g., 10 kb), including population differentiation index (Fst), nucleotide polymorphism (π), Tajima’s D, Fu and Li’s D. The selection signature of each window is further determined according to the calculated results. Based on the high-confidence consistent SNP data, the vcftools [27] software package was used to calculate all of the above population genetic parameters with the designated sliding window and step size settings.

### 2.5. Gene Enrichment Analysis and Reference Genome

Gene function annotation was performed using eggNOG-mapper [28]. The *M. armatus* OrgDB database was constructed using AnnotationForge (https://bioconductor.org/packages/release/bioc/html/AnnotationForge.html (accessed on 28 July 2021)). The reference genome of *M. armatus* was used in this study, which was a chromosome-level assembly generated by PacBio HiFi sequencing and Hi-C scaffolding. The genome size is approximately 600.1 Mb, with a Contig N50 of 9.9 Mb and 24 chromosomes assembled. This high-quality reference genome ensures high mapping rates and reliable variant detection in our resequencing analysis [15].

## 3. Results

### 3.1. Whole-Genome Resequencing Data Analysis

After quality control, a total of 209.05 Gbp of clean data was generated from the resequencing data of the three populations, yielding 697,876,043 clean reads. The Q30 value reached 94.42%, indicating a high sequencing quality. The average mapping rate against the reference genome was 97.85%, with an average coverage depth of 10×. The genome coverage was 91.40% (covered by at least one base) (Table 2). These results demonstrate that the sequencing data is of reliable quality and suitable for subsequent analyses.

### 3.2. SNP Detection and Statistical Annotation of Results

A total of 7,459,686 SNPs were detected on average across the three populations, including 4,510,018 transition SNPs and 2,949,668 transversion SNPs, with a transition/transversion ratio of 1.52. The number of heterozygous SNPs was 165,357 and that of homozygous SNPs was 7,294,329, accounting for a heterozygous SNP ratio of 2.22% (Table 3). The heterozygous SNP ratio was calculated as the number of heterozygous SNPs divided by the total number of detected SNPs across all individuals, rather than being counted at the individual genome level.

**Figure 1:**
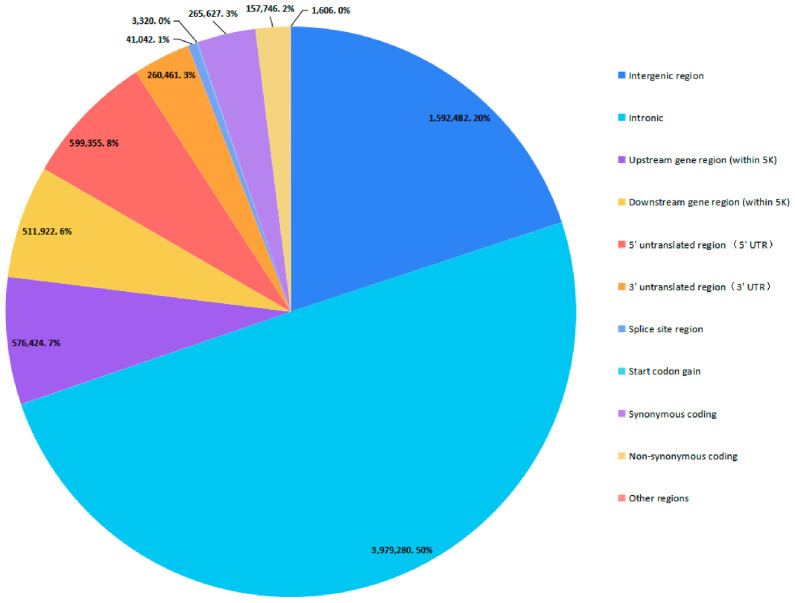
Functional annotation and classification of identified SNPs in three *M. armatus* populations.

Functional annotation of the identified SNPs from the three *M. armatus* populations revealed their distribution across distinct genomic regions, as detailed in Figure 1 and Table 4.

### 3.3. Detection and Result Annotation of Small Fragment Insertions and Deletions

The overall numbers of coding region insertions, coding region deletions, homozygous coding region indels, heterozygous coding region indels, total coding region indels, genome-wide insertions, genome-wide deletions, homozygous genome-wide indels, heterozygous genome-wide indels and total genome-wide indels detected in the three populations were 4402 ± 73, 5298 ± 88, 8788 ± 107, 913 ± 69, 9700 ± 159, 917,918 ± 11,690, 984,804 ± 11,582, 1,827,240 ± 17,482, 75,482 ± 9024 and 1,902,722 ± 23,247, respectively (Table 5).

### 3.4. Functional Annotation of Mutant Genes at the DNA Level

The GO classification systems of the samples from the three water areas were highly consistent. For cellular components (CCs), 17 categories were identified, ranging from the highly abundant cell/cell part (7796/7238) to the low-abundant nucleoid (1/1), with the categories and their corresponding gene numbers being completely identical across all samples. For molecular functions (MFs), 14 categories were found, dominated by binding (10,172/9552) and catalytic activity (5525/5251), and the gene numbers of specific functions such as toxin activity (1/1) were fixed. For biological processes (BPs), 23 categories were detected, with the core concentrated in basic processes including cellular process (8883/8409) and biological regulation (6265/5927), and consistent characteristics observed in low-abundant processes like cell aggregation (8/7).

Across all water area samples, the proportion of mutant genes in each GO category relative to the total genes was maintained at 85–95%, with no abnormally high or low variation detected in any functional gene set. For cellular components (CCs), the mutation proportion of membrane-related genes was approximately 93%. For molecular functions (MFs), the proportion of genes related to signal transducer activity was about 90%. For biological processes (BPs), the proportion of genes associated with response to stimulus reached roughly 95%. At the functional classification level, the GO annotation profiles of the three groups were highly consistent: 17 categories in cellular components, 14 in molecular functions, and 23 in biological processes. The gene counts in each subcategory were nearly identical. High-abundance functional categories—including cell/cell part, binding, catalytic activity, cellular process, and biological regulation—carried the majority of mutations and showed consistent variation patterns, while low-abundance or species-conserved functions (e.g., toxin activity, nucleoid) displayed identical total gene and mutant gene counts. For example, the variation proportion of membrane-related genes in cellular components was approximately 93% (membrane: 7374/6868; membrane part: 6605/6136); that of signal transducer activity genes in molecular functions was about 90% (1502/1358); and that of response to stimulus genes in biological processes was roughly 95% (3218/3051), which reflected a consistent variation pattern of core genes among the three populations.

All samples from the three water areas exhibited the characteristic of “active basic functions and conserved specific functions”. Specifically, active functions (e.g., binding, catalytic activity, and cellular processes) were supported by a large number of genes with stable variation, serving as the core underpinning for the life activities of the populations. In contrast, conserved functions (e.g., toxin activity, translational regulator activity, and nucleoid) showed identical numbers of total genes and mutant genes (e.g., 1/1 for toxin activity).

### 3.5. Genetic and Phylogenetic Analysis

The phylogenetic tree revealed that all *M. armatus* samples from Xunwu Water (XW), Jiuqu River (DN) and Taojiang River (XF) clustered within a single major phylogenetic clade, indicating a high level of species-level consistency among the *M. armatus* populations from the three water areas. These populations share a common ancestor, with no obvious species differentiation observed and close genetic relationships among them.

Some *M. armatus* samples from different water areas showed interleaved clustering in the phylogenetic tree and did not form independent clades strictly according to their water area origins. For instance, several clades containing samples from Xunwu Water and Taojiang River were closely adjacent or even interspersed.

**Figure 2:**
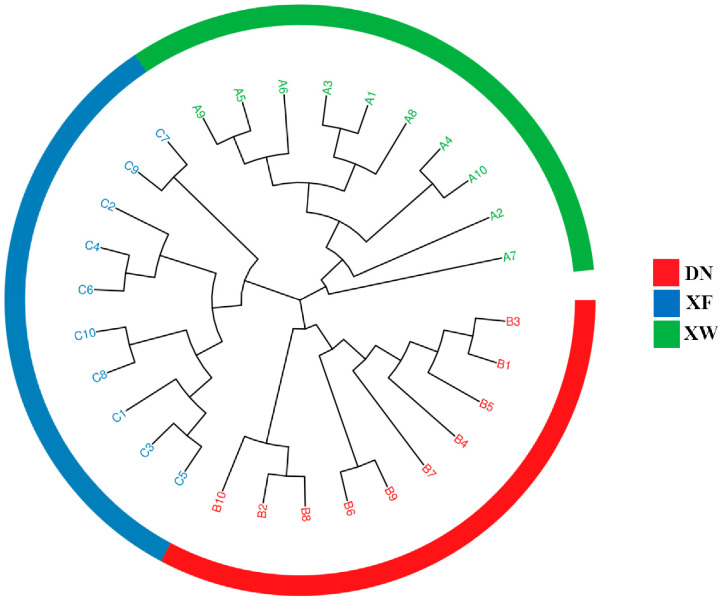
Neighbor-joining phylogenetic tree of the three *M. armatus* populations based on genome-wide SNPs. Individuals are color-coded by sampling site: red for DN (Jiuqu River, Dongjiang River source), blue for XF (Taojiang River, Ganjiang River source), and green for XW (Xunwushui, Dongjiang River source).

However, *M. armatus* samples from Jiuqu River exhibited a relatively concentrated clustering pattern and formed several relatively independent small clades. In contrast, samples from Xunwu Water and Taojiang River were distributed more dispersedly; although local relatively aggregated small clades were present in each population, the interspersion between them was more pronounced (see Figure 2 for details).

### 3.6. Population Genetic Structure and Diversity Analysis

**Figure 3:**
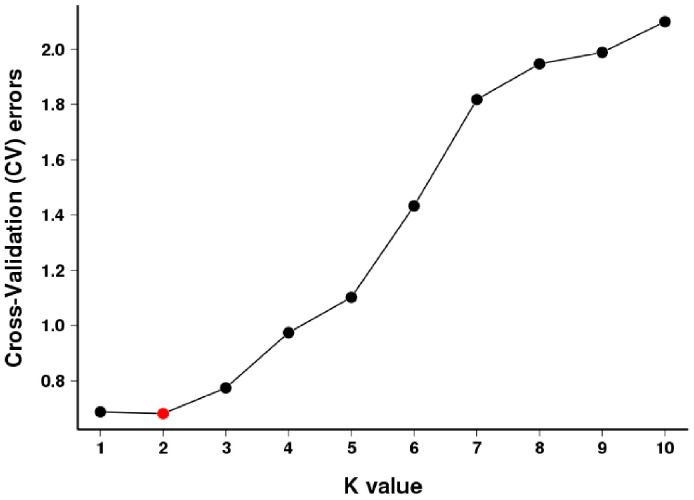
Cross-validation (CV) error plot for determining the optimal K value in population structure analysis using admixture. Cross-validation (CV) errors were calculated for each K value ranging from 1 to 10. The lowest CV error was observed at K = 2 (marked by the red dot), indicating that this value represents the optimal number of population clusters for the dataset.

**Figure 4:**
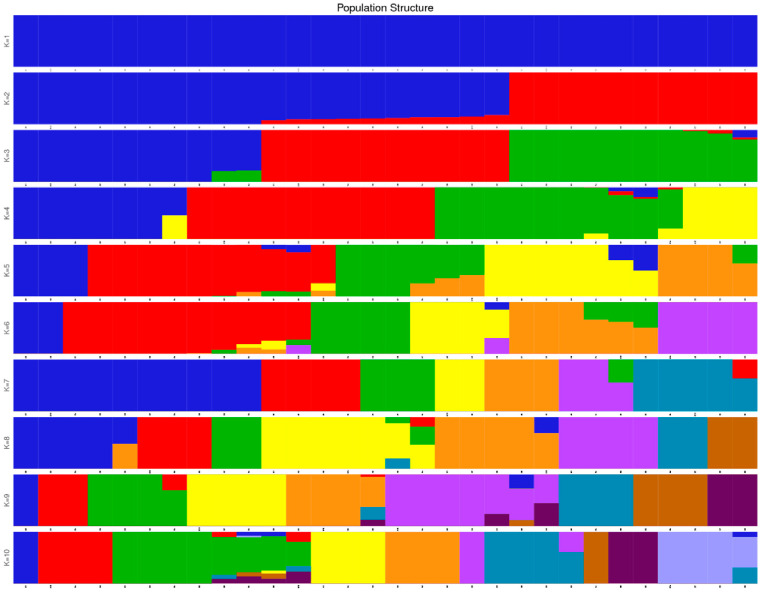
Sample clustering results corresponding to different K values.

Based on the cross-validation (CV) error curve from admixture analysis, the CV error reached a local minimum at K = 2 (marked by the red dot), indicating that the model provided the most reliable inference of the population genetic structure of *M. armatus* at this value, which is thus the core K for resolving the population structure (see Figure 3 for details). The genetic composition of *M. armatus* individuals from Xunwu Water (XW) and Jiuqu River (DN) was dominated by the blue component with an extremely low proportion of the red component, suggesting that these two populations had a genetic background highly biased toward one ancestral population, with strong intraspecific genetic consistency and significant genetic differentiation from the population of the other water area. In contrast, the genetic composition of Taojiang River (XF) individuals was dominated by the red component with a minimal blue component. Similar to XW and DN, the XF population was also highly biased toward a single ancestral population, but the ancestral population it leaned toward (red) differed from that of XW and DN (blue), indicating obvious genetic differentiation between XF and the other two populations. Notably, DN individuals exhibited a mixed pattern of blue and red genetic components, suggesting that the DN population harbored genetic contributions from both ancestral populations and represented the population with the highest level of genetic admixture (see Figure 4 for details).

**Figure 5:**
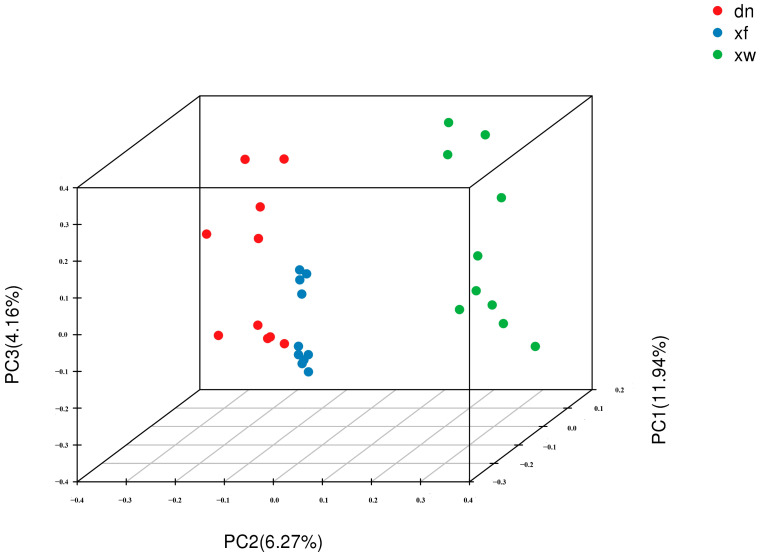
Three-dimensional principal component analysis (PCA) plot of the three populations based on SNP data. The plot visualizes the genetic relationships among individuals from three populations: red for DN (Jiuqu River, Dongjiang River source), blue for XF (Taojiang River, Ganjiang River source), and green for XW (Xunwushui, Dongjiang River source). The first three principal components (PC1, PC2, and PC3) explain 11.94%, 6.27%, and 4.16% of the total genetic variance, respectively. Clear genetic separation is observed among the three populations, indicating distinct population structures.

From the clustering results of principal component analysis (PCA), PC1 (≈11.94%), PC2 (≈6.27%) and PC3 (≈4.16%) collectively explained approximately 22.37% of the total genetic variation. Although not covering all variation, these principal components reflected the major patterns of genetic differentiation among populations. The XW, DN and XF populations all showed strong intraspecific genetic consistency but significant interspecific genetic differentiation, each forming an independent genetic cluster. Genetic differentiation between DN and XF was bidirectional and complete; XW had a relatively close genetic distance to DN but significant differentiation from XF (see Figure 5 for details).

**Figure 6:**
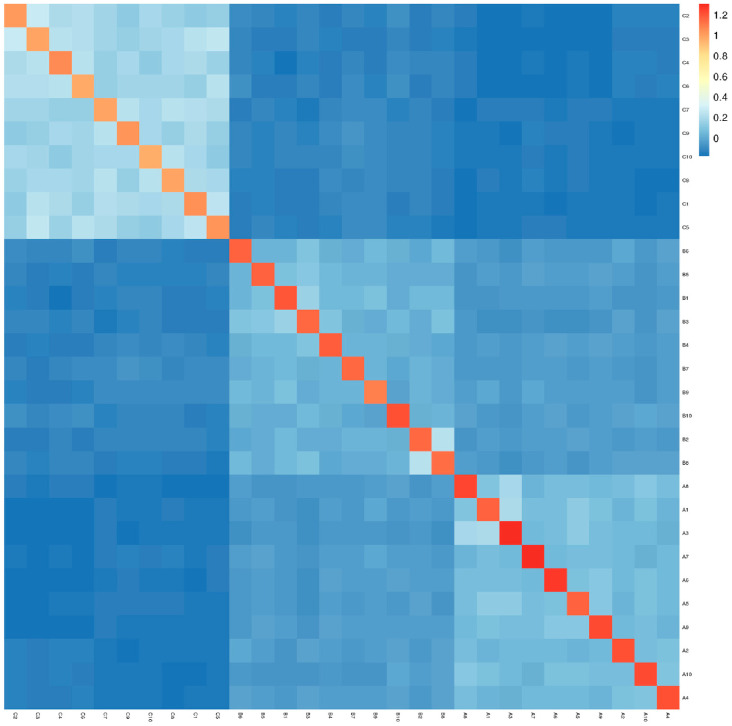
Heatmap of pairwise genetic relatedness among individuals from three populations. The heatmap visualizes the genetic relatedness coefficients calculated from genome-wide SNP data. The color gradient from blue to red represents increasing relatedness (range: 0–1), with redder cells indicating higher genetic similarity. Individuals are grouped by their population origin, revealing strong within-population relatedness (diagonal) and low genetic relatedness between populations.

A heatmap based on kinship values was used to compare the genetic relatedness of *M. armatus* populations from the three water areas (XW: Series A; DN: Series B; XF: Series C). Kinship values reflect the degree of genetic relatedness between individuals, with values closer to 1 indicating closer relatedness and values closer to 0 indicating greater genetic distance. In the heatmap, kinship values among XW individuals showed a cluster of medium-to-high values (orange-red in color); for example, the kinship values between A1 and A2, A3 and other XW individuals were significantly higher than those between XW individuals and individuals from other water areas. This indicated close genetic relatedness within the XW population, strong intraspecific genetic consistency, and potential inbreeding or sufficient genetic exchange within the small population. Kinship values among DN individuals also showed a concentration of medium-to-high values (orange-red in color), with high values between B1 and B2, B3 and other DN individuals, suggesting close relatedness within the DN population and a relatively stable and tightly linked internal genetic structure. Kinship values among XF individuals were dominated by medium-to-low values (blue in color); the kinship values between C1 and C2, C3 and other XF individuals were significantly lower than those within the XW and DN populations, reflecting relatively distant genetic relatedness within the XF population and richer genetic diversity at the individual relatedness level. Kinship values between XW (Series A) and DN (Series B) individuals were generally in the medium-to-low range (blue in color), indicating distant genetic relatedness and significant genetic differentiation between the two populations, which may result from restricted genetic exchange due to geographic isolation or ecological environmental differences. Kinship values between XW (Series A) and XF (Series C) individuals were also dominated by medium-to-low values (blue in color), suggesting distant relatedness and obvious differences in genetic background between these two populations. Similarly, kinship values between DN (Series B) and XF (Series C) individuals fell in the medium-to-low range (blue in color), reflecting loose genetic relatedness and prominent genetic differentiation between the DN and XF populations (see Figure 6 for details).

**Figure 7:**
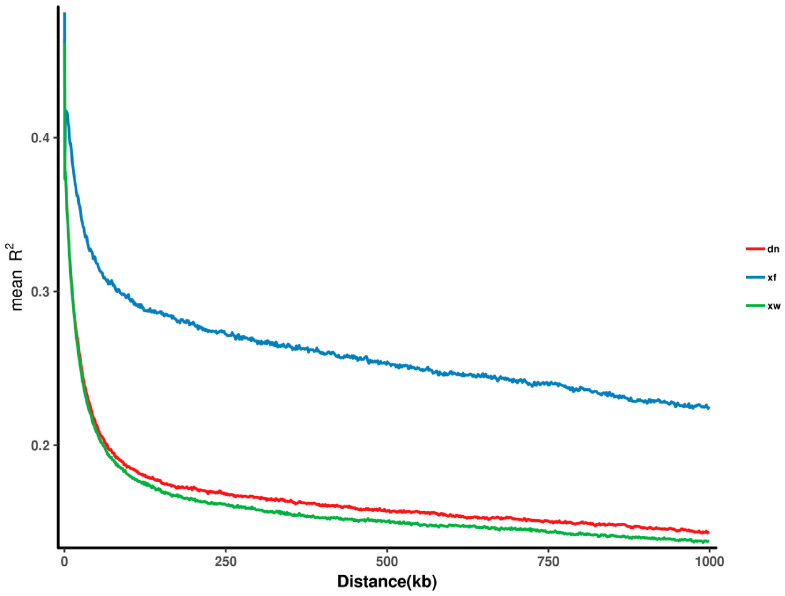
Linkage disequilibrium (LD) decay patterns in the three populations. LD decay is measured as the squared correlation coefficient r^2^ between pairs of SNPs, plotted against physical distance (kb) across the genome. The blue, red, and green lines represent the DN (Jiuqu River, Dongjiang River source), XF (Taojiang River, Ganjiang River source), and XW (Xunwushui, Dongjiang River source) populations, respectively. LD decays with increasing physical distance in all populations, with the XF population showing the slowest decay rate, indicating higher levels of long-range linkage disequilibrium compared to the other two populations.

Based on the linkage disequilibrium (LD) decay curves (mean R^2^ vs. physical distance) of *M. armatus* from the three water areas (XW, DN, and XF), all three curves showed a trend of a rapid decrease in mean R^2^ with increasing physical distance followed by a gradual plateau, consistent with the universal law of genomic LD decay: the shorter the physical distance between loci, the higher the level of LD; the longer the distance, the more easily recombination events break the linkage, leading to lower LD. The XF population had the highest initial mean R^2^ value and the slowest decay rate within the short distance range (0–100 kb), with its curve consistently at the top. The DN population had a lower initial mean R^2^ than XF, with a decay rate faster than XF but slower than XW within 0–100 kb, and its curve lay between those of XF and XW. The XW population had the lowest initial mean R^2^ and the fastest decay rate within 0–100 kb, with its curve consistently at the bottom (see Figure 7 for details). Neutrality tests (Tajima’s D and Fu and Li’s D) were performed to assess the demographic history of the three populations. None of the tests showed statistically significant deviation from neutral expectations (*p* > 0.05). The XW and DN populations had positive Tajima’s D values (0.16 and 0.36, respectively) and positive Fu and Li’s D values (2.97 and 3.44, respectively). The Xinfeng (XF) population had negative values for both Tajima’s D (−0.67) and Fu and Li’s D (−2.63).

The nucleotide diversity (π) of the XW (0.00490 ± 0.00248) and DN (0.00478 ± 0.00312) populations remained at relatively high levels, while the π value of the XF (0.00463 ± 0.00158) population was significantly lower. The lowest FST value (0.12) was observed between XW and Dingnan (DN) populations. The XF population exhibits the highest differentiation from the other two groups, with FST values of 0.19 vs. XW and 0.17 vs. DN. All populations had an average minor allele frequency (Average_MAF) in the range of 0.26–0.28 without extreme high or low values, indicating a relatively balanced distribution of alleles within each population and no overdominance of a single allele. The values of expected heterozygosity and observed heterozygosity were close in all populations (e.g., DN: 0.3211 vs. 0.3213; XW: 0.3185 vs. 0.3088), with no significant deviation. A large number of polymorphic markers were detected in all three populations (Number_of_poly_marker > 340,000 for all), and the polymorphism information content (PIC) ranged from 0.20 to 0.26, representing a moderate level of polymorphism. The three populations could be clearly divided into a high-diversity group (DN and XW) and a low-diversity group (XF). The DN and XW populations showed highly similar values in all eight core diversity indicators, all of which were significantly higher than those of the XF population. Both populations had over 440,000 polymorphic markers (DN: 448,746; XW: 446,349), an observed number of alleles close to 1.92 (DN: 1.922253; XW: 1.917326), and an expected number of alleles of approximately 1.54 (DN: 1.5482; XW: 1.5433). Both populations had an expected heterozygosity > 0.31 (DN: 0.3211; XW: 0.3185), a Nei’s diversity index > 0.33 (DN: 0.3387; XW: 0.336), a Shannon–Wiener index > 0.47 (DN: 0.4815; XW: 0.4778), and a PIC of approximately 0.25 (DN: 0.2572; XW: 0.2552), with all indices at a relatively high level. For the low-diversity group (XF), all core diversity indicators of the XF population were significantly lower than those of DN and XW. XF had only 342,646 polymorphic markers (≈106,000 fewer than DN), an observed number of alleles of 1.704198 (0.218 lower than DN), and an expected number of alleles of 1.4529 (0.095 lower than DN). XF had an expected heterozygosity of 0.2608 (0.0603 lower than DN), a Nei’s diversity index of 0.2752 (0.0635 lower than DN), a Shannon–Wiener index of 0.3864 (0.0951 lower than DN), and a PIC of 0.2073 (0.0499 lower than DN), with all indices at the lowest level among the three populations. Notably, the Average_MAF of XF (0.2829) was slightly higher than that of DN (0.2616) and XW (0.2606), which was the only indicator that did not follow the low-diversity pattern (see Table 6 for details).

## 4. Discussion

Studies on Megalobrama have confirmed that geographic isolation and reduced gene flow are primary drivers of population differentiation, with isolated populations showing lower genetic diversity, stronger genetic structure, and elevated linkage disequilibrium [29]. Consistent with these patterns, our results for *M. armatus* reveal clear genetic differentiation shaped by river basin geography. Nucleotide diversity (π) was high in the Xunwu (XW, 0.00490 ± 0.00248) and Dingnan (DN, 0.00478 ± 0.00312) populations but significantly lower in the Xinfeng (XF, 0.00463 ± 0.00158) population. Pairwise FST values indicate moderate differentiation between XW and DN (FST = 0.12), consistent with their shared Dongjiang River origin, while the highest divergence was observed between XF and the other two populations (XF vs. XW: 0.19; XF vs. DN: 0.17), reflecting strong genetic isolation in the Ganjiang River system. Neutrality tests (Tajima’s D and Fu and Li’s D) showed no significant deviations from neutrality (*p* > 0.05). Positive values in XW (Tajima’s D = 0.16; Fu and Li’s D = 2.97) and DN (Tajima’s D = 0.36; Fu and Li’s D = 3.44) suggest stable or slightly contracting populations, while negative values in XF (Tajima’s D= −0.67; Fu and Li’s D = −2.63) hint at population expansion, though this is not statistically significant. Despite this clear genetic structure, GO enrichment analysis revealed no significant functional genetic differentiation among the three populations. The Xunwu and Jiuqu Rivers (XW and DN) share high connectivity, supporting gene flow and moderate differentiation. The Tao River (XF), though geographically isolated by watershed barriers, experiences similar environmental conditions (water temperature, quality, and food resources) to the other two systems, resulting in weak divergent selection and limited functional divergence. To further characterize adaptive and demographic processes, future work should focus on environment-related genes and their expression patterns, as well as the effects of fine-scale habitat heterogeneity on population divergence. For conservation, priority should be given to in situ habitat protection and natural recovery to maintain genetic diversity. If intervention is needed, artificial gene flow measures must be implemented cautiously, with formal risk assessment and monitoring to avoid disrupting local adaptive genetic backgrounds.

Relevant studies on the population genetic differentiation of aquatic animals have fully confirmed the close correlation between species genetic structure and geographical distribution, breeding models, and habitat characteristics. Studies on *Scylla paramamosain* have shown that it exhibits the characteristics of low polymorphism, weak population differentiation, and low inbreeding levels; the results of PCA, admixture grouping (K = 1–5), phylogenetic tree, and LD decay analysis are highly consistent, all indicating no significant genetic differentiation among relevant geographical populations [30]. Studies on the genetic pattern of Channa argus in Shandong have confirmed that its genetic clustering strictly matches geographical distribution, with geographical isolation being the core natural factor for differentiation; wild populations in independent water areas such as the Yellow River Estuary and Dongping Lake have formed distinct genetic clades, while populations in Nansi Lake and Yihe River have closer genetic relationships due to high aquatic connectivity [31]. Relevant studies on *Megalobrama amblycephala* have identified the genetic structure and selection signals of different geographical populations, confirming their independent ancestral components and limited gene flow among different geographical populations, with geographical isolation as the dominant factor for differentiation, which has laid a genomic foundation for the classification and protection of its germplasm resources [32]. Studies have found that the genetic diversity of farmed populations of *Patinopecten yessoensis* in China is significantly lower than that of wild populations, with a tendency toward homogeneous genetic structure and significant risks of germplasm degradation [33].

In research on stress-resistant breeding of aquatic animals, candidate genes and QTLs associated with heat tolerance have been identified in *Lateolabrax maculatus*, and QTLs for low-temperature tolerance have been detected in *Chlamys farreri*, which provides a genomic basis for temperature stress-resistant breeding of aquatic organisms. Meanwhile, certain QTLs in hybrid scallops have been found to exhibit overdominance effects, offering a genetic foundation for developing novel cold-tolerant and fast-growing strains by exploiting interspecific heterosis [34,35]. In the field of genomic selection breeding, machine learning-based whole-genome feature selection strategies have shown excellent application value: they can accurately screen 3–6% of core loci from 8.22 million SNPs in sturgeon, improving the genomic prediction accuracy of core economic traits such as caviar yield, color, and body weight by 3.4–4.6% [36].

Classical studies employing phylogenetic trees, PCA and admixture analyses have confirmed that geographical isolation is the core driving factor for population differentiation in freshwater fish. Wild populations of *Percocypris pingi* have formed multiple genetically differentiated units according to their geographical distribution, which is consistent with the findings of studies on *Megalobrama* and *Channa argus* [37]. Meanwhile, whole-genome resequencing technology has been applied to research on the dispersal of downstream *Coilia brachygnathus* into large reservoirs. The dispersed populations exhibit moderate levels of nucleotide diversity (π) and heterozygosity (He), a short LD decay distance, and a recent rapid increase in effective population size (Ne), which conform to the typical genetic characteristics of dispersed species—rapid proliferation after a bottleneck effect [38].

Multi-dimensional analyses of *M. armatus* from the three major water systems in Ganzhou in this study revealed a unique genetic pattern. The phylogenetic tree showed that all samples from the three locations belong to the same evolutionary clade, with a high degree of consistency at the species level, a common ancestor, no obvious speciation, and overall close genetic relationships. Among them, samples from the Jiuqu River were clustered closely and formed an independent small clade with significant genetic uniqueness, which may be attributed to the stable ecological environment and certain geographical isolation of this water system, leading to the accumulation of specific genetic variations during evolution; samples from the Xunwu River and Tao River were scattered in distribution, with obvious interspersion despite local clustering, reflecting more frequent gene flow between their populations, possibly due to weak selective pressure from the ecological environment resulting in insignificant genetic differences among populations.

Admixture population structure analysis revealed that the model exhibited the highest goodness of fit at K = 2, confirming that *M. armatus* from the three river systems derive from two distinct ancestral populations. The Taojiang River (XF) population showed nearly pure ancestry from the red ancestral component, while the Xunwushui (XW) population was dominated by the blue ancestral component. In contrast, the Jiuqu River (DN) population exhibited clear admixture of both ancestral components, with intermediate ancestry proportions between XW and XF. This pattern supports a transitional or “genetic bridge” status for DN, potentially reflecting its role in facilitating gene flow between the two geographically separated populations. To further validate this hypothesis, formal tests of introgression (e.g., D-statistics/ABBA-BABA tests, f-statistics, and coalescent modeling) are recommended in future work to quantitatively assess the magnitude, directionality, and timing of gene flow among the three populations.

PCA analysis further revealed that the Jiuqu River formed an independent cluster in the core genetic dimension, with a high degree of differentiation from the Tao River and Xunwu River; the Tao River exhibited a unique genetic variation pattern, with bidirectional genetic differentiation from the other two water systems; the Xunwu River and Jiuqu River had partially adjacent distribution areas, with a short genetic distance and closer connections, but significant differentiation from the Tao River. Kinship heatmap analysis indicated that within populations, individuals from the Xunwu River and Jiuqu River showed aggregation of medium-to-high kinship values, with close genetic relationships and strong genetic consistency, which may be related to inbreeding within populations and sufficient gene flow in small populations; individuals from the Tao River had predominantly medium-to-low kinship values, with distant genetic relationships and richer genetic diversity at the individual kinship level. Among populations, kinship values among the three locations were all medium to low, with no close genetic relationships and prominent differentiation characteristics, which may be caused by restricted gene flow due to geographical isolation and differences in ecological environments. LD decay distance analysis showed that the Tao River had the longest LD decay distance, suggesting a small population size, strong genetic drift and low recombination rate; the Jiuqu River had a moderate LD decay distance, with intermediate levels of effective population size and recombination rate; the Xunwu River had the shortest LD decay distance, indicating a large population size, weak genetic drift and frequent recombination, which can quickly break linked allelic combinations. Population genetic diversity analysis showed that all three locations conform to the regular patterns of normal freshwater fish, without being affected by severe inbreeding drift, with stable genetic structures. More than 340,000 polymorphic markers were detected in all populations, providing a solid foundation for subsequent research. Among populations, they can be divided into a high-diversity group (Jiuqu River and Xunwu River) and a low-diversity group (Tao River). The former had significantly higher core indicators and richer genetic variations; the latter had more homogeneous genomic variant loci and allelic types, with significantly lower diversity. Only the Tao River had a slightly higher Average_MAF, due to the high minor allele frequency at a small number of variant loci, presenting a special characteristic of locally high-frequency variation but overall low diversity.

In summary, the *M. armatus* populations from the three major water systems of Xunwu River, Jiuqu River and Tao River in the Ganzhou water system exhibit strong genetic consistency within each population, each forming an independent genetic cluster, with significant genetic differentiation among populations. Among them, the Jiuqu River and Tao River populations show bidirectional and complete genetic differentiation; the Xunwu River and Jiuqu River populations have a short genetic distance, while the Xunwu River and Tao River populations exhibit significant genetic differentiation. Differences in LD decay distances reflect the divergent characteristics of the three populations in terms of effective population size and recombination rate: the Tao River population is characterized by a small population size and low recombination rate, the Xunwu River population by a large population size and high recombination rate, and the Jiuqu River population by intermediate characteristics between the two.

Based on the results of this study, germplasm resource conservation and research on *M. armatus* can be carried out from four aspects in the follow-up. First, aiming at the genetic correlation between the Xunwu River and Jiuqu River populations, investigate the ecological connectivity (river course connectivity, migratory behavior, etc.) of the two water systems, explore the ecological driving factors of their genetic correlation, and verify the impact of effective population size on LD decay distance combined with population ecological surveys. Second, systematically investigate the ecological environmental factors of the three water systems, analyze the shaping effect of ecological environmental heterogeneity on population genetic structure, and provide a scientific basis for habitat conservation. Third, establish a long-term population dynamic monitoring mechanism to track changes in genetic structure and gene flow, adjust conservation and management strategies in a timely manner, and maintain genetic diversity and ecological balance. Fourth, formulate differentiated conservation strategies based on our findings. Given the significantly lower genetic diversity and distinct genetic structure of the Taojiang River (XF) population, priority should be given to strengthening habitat protection, reducing human disturbance, and preventing habitat fragmentation to effectively curb the further loss of genetic diversity [39]. For the Xunwushui (XW) and Jiuqu River (DN) populations, which exhibit high genetic similarity and a risk of inbreeding, long-term monitoring of population status and genetic structure is essential. When necessary, appropriate artificial gene flow strategies can be implemented to enhance genetic exchange, maintain long-term genetic health, and preserve the evolutionary potential of local populations [40].

## 5. Conclusions

All three populations belong to the same evolutionary clade and originate from two ancestral components. The XW and DN populations show high genetic diversity, close genetic relationship and short linkage disequilibrium decay distance, while the XF population exhibits low genetic diversity, distant individual relatedness and long linkage disequilibrium decay distance. Significant genetic differentiation exists among populations, which is mainly driven by geographical isolation and limited gene flow between river systems. These findings provide genome-level insights into the genetic background and differentiation mechanism of *M. armatus* in southern river systems. The results support the development of molecular markers, the analysis of environmental adaptation mechanisms, and the formulation of targeted conservation strategies. Based on our whole-genome resequencing analysis of *M. armatus* from the Dongjiang and Ganjiang River sources, we confirm that the XF (Taojiang River) population shows significantly lower genetic diversity, longer LD decay distance, and distinct genetic differentiation, while the XW (Xunwushui) and DN (Jiuqu River) populations have high genetic diversity but face potential inbreeding risks due to limited gene flow and strong intra-population genetic consistency. To maintain the long-term genetic health and sustainable utilization of this valuable freshwater fish resource, we propose the following priority actions and practical strategies: Strengthen targeted conservation for the XF population. Establish in situ conservation zones in the Taojiang River to protect spawning and feeding habitats, reduce human disturbance, overfishing, and water pollution. Implement ex situ conservation programs, including captive breeding stocks using genetically distinct XF individuals to avoid further diversity loss. Conduct regular monitoring of genetic diversity indices (He, PIC, and MAF) to track recovery trends. Monitor and mitigate inbreeding risks in XW and DN populations. Carry out genetic health screening using genome-wide SNP markers to identify individuals with high homozygosity and potential inbreeding. Promote moderate artificial gene flow between subpopulations within the Dongjiang source system to enhance genetic exchange and reduce genetic uniformity. Set up long-term population monitoring plots to survey population size, age structure, and recruitment dynamics. Restore hydrological connectivity and reduce geographic isolation. Remove or retrofit man-made barriers (small dams and weirs) to restore natural migration routes and facilitate natural gene flow between river systems. Protect watershed vegetation and maintain ecological flow to stabilize aquatic habitats and reduce selection pressure on local populations. Establish a genomic monitoring and management platform. Develop a panel of diagnostic SNP markers for rapid identification of genetic origin, diversity, and differentiation. Build a database integrating genetic data, population surveys, and environmental factors to support dynamic conservation decision-making. Enforce fishery management and policy support. Strictly enforce closed fishing seasons and minimum capture size regulations for wild *M. armatus*. Promote standardized artificial breeding using genetically superior broodstock to reduce fishing pressure on wild populations.

## Figures and Tables

**Table 1:** Sample information of the *M. armatus* populations.

Population	Latitude	Longitude	Drainage	River
Xunwu (XW)	25.0085	115.7439	Headwaters of the Dongjiang River	Xunshui River
Dingnan (DN)	24.9129	115.2231	Headwaters of the Dongjiang River	Jiuqu River
Xinfeng (XF)	25.4649	114.9815	Headwaters of the Ganjiang River	Taojiang River

**Table 2:** Quality and alignment summary of whole genome resequencing data.

Population	Bases/bp	GC Content/%	Q20/%	Q30/%	Reads	Alignment Rate/%	Sequencing Depth
XW	70,075,162,040	40.23	97.90	94.36	233,938,669	97.85	10.20
DN	72,130,154,974	40.23	97.94	94.42	240,800,045	97.86	10.30
XF	66,844,317,454	40.21	97.95	94.49	223,137,329	97.84	9.70

**Table 3:** Statistics of single nucleotide polymorphisms (SNPs) obtained from sequence detection.

Population	SNP Number	SNP Number (Transition)	SNP Number (Transversion)	Transition/Transversion SNP Ratio	SNP Number (Heterozygous)	SNP Number (Homozygous)	Heterozygous SNP Percentage (%)
XW	7,467,441 ± 61,589 ^ab^	4,514,787 ± 37,776 ^ab^	2,952,654 ± 23,815 ^ab^	1.52 ± 0 ^a^	173,325 ± 10,282 ^a^	7,294,117 ± 53,593 ^a^	2.32 ± 0.1476 ^a^
DN	7,483,236 ± 64,472 ^a^	4,524,336 ± 39,454 ^a^	2,958,900 ± 25,021 ^a^	1.52 ± 0 ^a^	180,267 ± 10,456 ^a^	7,302,968 ± 55,468 ^a^	2.43 ± 0.1252 ^a^
XF	7,428,382 ± 45,245 ^b^	4,490,931 ± 27,732 ^b^	2,937,451 ± 17,517 ^b^	1.52 ± 0 ^a^	142,478 ± 7749 ^b^	7,285,903 ± 43,635 ^a^	1.91 ± 0.0994 ^b^
Total	7,459,686 ± 60,435	4,510,018 ± 36,997	2,949,668 ± 23,441	1.52 ± 0	165,357 ± 19,090	7,294,329 ± 49,873	2.22 ± 0.2578

Note: Data are presented as the mean ± standard deviation. Different superscript letters in the same column indicate significant differences at the level of *p* < 0.05.

**Table 4:** Functional annotation statistics of single nucleotide polymorphisms (SNPs) identified by sequence detection.

Population	Intergenic Region	Intron	Upstream Gene Region (Within 5 K)	Downstream Gene Region (Within 5 K)	5′ Untranslated Region (5′ UTR)	3′ Untranslated Region (3′ UTR)	Splice Acceptor Site	Splice Donor Site
XW	1,594,206 ± 13,286 ^ab^	3,983,366 ± 32,141 ^ab^	577,187 ± 4910 ^ab^	512,281 ± 4234 ^ab^	60,066 ± 555 ^ab^	260,706 ± 1848 ^ab^	393 ± 6 ^a^	384 ± 8 ^b^
DN	1,597,961 ± 13,795 ^a^	3,991,511 ± 33,812 ^a^	578,180 ± 5018 ^a^	513,580 ± 4421 ^a^	60,145 ± 641 ^a^	261,191 ± 1863 ^a^	396 ± 8 ^a^	390 ± 5 ^a^
XF	1,585,280 ± 9677 ^b^	3,962,964 ± 23,565 ^b^	573,904 ± 3636 ^b^	509,905 ± 3075 ^b^	59,595 ± 482 ^b^	259,487 ± 1304 ^b^	390 ± 5 ^a^	383 ± 5 ^b^
Total	1,592,482 ± 13,121	3,979,280 ± 31,573	5764,24 ± 4780	511,922 ± 4118	599,355 ± 597	260,461 ± 1788	393 ± 7	386 ± 7
Population	Splice site region	Start codon gain	Start codon loss	Synonymous coding	Non-synonymous coding	Synonymous stop codon	Stop codon gain	Stop codon loss
XW	41,096 ± 408 ^a^	3330 ± 28 ^a^	229 ± 2 ^a^	265,892 ± 2762 ^a^	158,011 ± 1569 ^ab^	199 ± 2 ^ab^	271 ± 5 ^a^	126 ± 2 ^a^
DN	41,185 ± 425 ^a^	3328 ± 35 ^a^	231 ± 3 ^a^	266,604 ± 2883 ^a^	158,358 ± 1707 ^a^	202 ± 4 ^a^	272 ± 6 ^a^	127 ± 2 ^a^
XF	40,846 ± 293 ^a^	3302 ± 27 ^a^	230 ± 4 ^a^	264,386 ± 2025 ^a^	156,868 ± 1200 ^b^	198 ± 3 ^b^	267 ± 7 ^a^	127 ± 2 ^a^
Total	41,042 ± 395	3320 ± 32	230 ± 3	265,627 ± 2665 ^a^	157,746 ± 1592	200 ± 3	270 ± 6	127 ± 2

Note: Data are presented as the mean ± standard deviation. Different superscript letters in the same column indicate significant differences at the level of *p* < 0.05.

**Table 5:** Statistics of insertions and deletions in the whole genome and coding regions.

Population	Coding Region Insertions	Coding Region Deletions	Homozygous Indels in Coding Regions	Heterozygous Indels in Coding Regions	Total Indels in Coding Regions	Whole -Genome Insertions	Whole -Genome Deletions	Homozygous Indels in Whole Genome	Heterozygous Indels in Whole Genome	Total Indels in Whole Genome
XW	4400 ± 72 ^a^	5295 ± 84 ^a^	8776 ± 106 ^a^	919 ± 53 ^a^	9695 ± 154 ^a^	918,905 ± 12,529 ^a^	986,020 ± 11,990 ^a^	1,826,216 ± 19,229 ^a^	78,709 ± 5940 ^a^	1,904,925 ± 24,504 ^a^
DN	4426 ± 85 ^a^	5329 ± 101 ^a^	8793 ± 125 ^a^	962 ± 65 ^a^	9755 ± 184 ^a^	921,581 ± 12,935 ^a^	989,080 ± 12,719 ^a^	1,828,376 ± 19,543 ^a^	82,285 ± 6392 ^a^	1,910,661 ± 25,638 ^a^
XF	4380 ± 60 ^a^	5270 ± 75 ^a^	8794 ± 98 ^a^	856 ± 44 ^b^	9650 ± 132 ^a^	913,268 ± 8720 ^a^	979,312 ± 8438 ^a^	1,827,127 ± 15,182 ^a^	65,453 ± 3387 ^b^	1,892,580 ± 17,142 ^a^
Total	4402 ± 73	5298 ± 88	8788 ± 107	913 ± 69	9700 ± 159	917,918 ± 11,690	984,804 ± 11,582	1,827,240 ± 17,482	75,482 ± 9024	1,902,722 ± 23,247

Note: Data are presented as the mean ± standard deviation. Different superscript letters in the same column indicate significant differences at the level of *p* < 0.05.

**Table 6:** Population genetic diversity.

Population	Average_MAF	Expected_Allele_Number	Expected_Heterozygous_Number	Nei_Diversity_Index	Number_of_Poly_Marker	Observed_Allele_Number	Observed_Heterozygous_Number	Polymorphysm_Information_Content	Shannon_Wiener_Index
XW	0.2606	1.5433	0.3185	0.336	446,349	1.917326379	0.3088	0.2552	0.4778
DN	0.2616	1.5482	0.3211	0.3387	448,746	1.922252639	0.3213	0.2572	0.4815
XF	0.2829	1.4529	0.2608	0.2752	342,646	1.704198316	0.2581	0.2073	0.3864

## Data Availability

The raw data supporting the conclusions of this study are available from the corresponding authors upon reasonable request for academic research purposes.
